# New white light-emitting halochromic stilbenes with remarkable quantum yields and aggregation-induced emission

**DOI:** 10.1038/s41598-022-06435-w

**Published:** 2022-02-11

**Authors:** Farhad Panahi, Ali Mahmoodi, Sajjad Ghodrati, Ali Ashtiani Abdi, Fazlolah Eshghi

**Affiliations:** 1 Chemistry Department, College of Sciences, Shiraz University, 71454 Shiraz, Iran; 2 Department of Polymer Engineering and Color Technology, Amirkabir University of Technology, Tehran, Iran; 3 Department of Organic Colorants, Institute for Color Science and Technology, Tehran, Iran

**Keywords:** Chemistry, Optics and photonics

## Abstract

Highly efficient single-component white light emitters (SWLEs), are attractive candidates for the simple and cost-effective fabrication of high-performance lighting devices. This study introduced a donor–π–acceptor and a donor–π–donor stilbene-based chromophores, representing pH-responsive fluorescence. The emitters showed yellow and green fluorescence in their neutral form. At the same time, protonation of the chromophores caused blue fluorescence color with a strong hypsochromic shift. The white light emission (WLE) for these chromophores was observed at approximately pH  3 due to the simultaneous presence of the neutral and protonated forms of the chromophores, covering almost all the emission spectra in the visible region (400–700 nm). These chromophores presented exceptional white light quantum yields (Φ) between 31 and 54%, which was desirable for producing white light-emitting devices. Density functional theory (DFT) and time-dependent (TD)-DFT were applied to study the structural and electronic properties of the chromophores.

## Introduction

**Figure 1:**
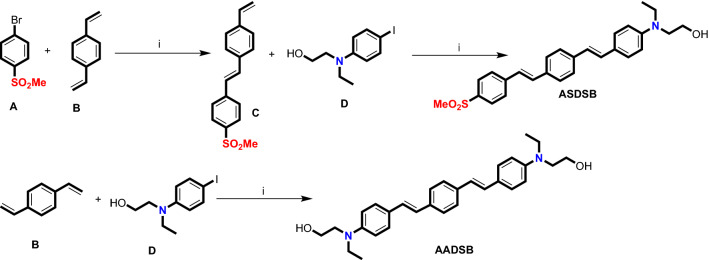
The chemical structure of halochromic stilbene-based chromophores and the synthetic pathway for their preparation. (i) Pd(OAc)_2_, DPEPhos, K_2_CO_3_, DMF, Ar.

Light sources based on white organic light-emitting diodes (WOLEDs), owing to their high efficiency and flexibility, low final price, long lifespan, and good color quality have emerged as a powerful platform to replace the traditional lighting devices such as the incandescent bulb, fluorescent lamps, and inorganic light-emitting diodes (LED)^1–4^. Conventional white light emission (WLE) in WOLEDs is obtained by combining three primary red, green, and blue emitters or two complementary-color emitters (for example, a yellow and a blue emitter)^5–7^. However, employing multiple emitters in fabricating WOLEDs is usually accompanied by several challenges such as complicated fabrication methods. Moreover, it is highly problematic to control the emission hue due to the energy transfer between different emitters. Additionally, the difference in photo-stability of emitters causes hue alteration in the long-term application of lighting devices^8–12^. Employing single-component white light emitters (SWLEs) whose emission spectra cover the whole visible region ranging from 400 to 700 nm can be an exceptional alternative for the simple fabrication of WOLEDs without the previously mentioned drawbacks. The sole structure of single-component white light emitters (SWLEs) can eliminate charge transfer and stability problems. Moreover, SWLE-based lighting devices can enjoy lower fabrication costs and less driving voltage than traditional lighting sources^13–15^. White fluorescence can be obtained through different photochemical procedures such as excited-state intramolecular proton transfer (ESIPT)^16–19^, aggregation-induced emission (AIE)^20–22^, halochromism^22–30^ or their combinations^31–33^. Halochromism is a specific type of ionochromismin which a change in pH value can induce the color of a fluorescent compound. White light emission can be generated from pH-dependent fluorescent dyes at a specific pH where the emission of neutral and protonated forms is complementary and identical. The synthesis of halochromic chromophores is usually straightforward because these compounds are comparatively small molecules with a simple chemical structure^34^. Moreover, tunable fluorescent devices can be easily fabricated by a single halochromic chromophore through pH regulation instead of using multiple emitters in separate layers. They also benefit from better color fastness during multiple voltage cycles and prolonged application time^35^. Some other applications of halochromic fluorescent chromophores include pH-sensitive sensors, biomedical probes, and textile dyes^36–44^. However, only a limited number of halochromic white light emitters have been synthesized so far, and the low quantum yield is still a critical issue. Herein, in continuation of our previous reports^45–50^, two highly efficient donor–π–acceptor and donor–π–donor stilbene-based fluorescent chromophores (Fig. 1) with white light emission introduced and their photophysical properties investigated.

## Results and discussion

Compounds **ASDSB** and **AADSB** were synthesized based on our previously reported procedure in the literature^51^.

**Figure 2:**
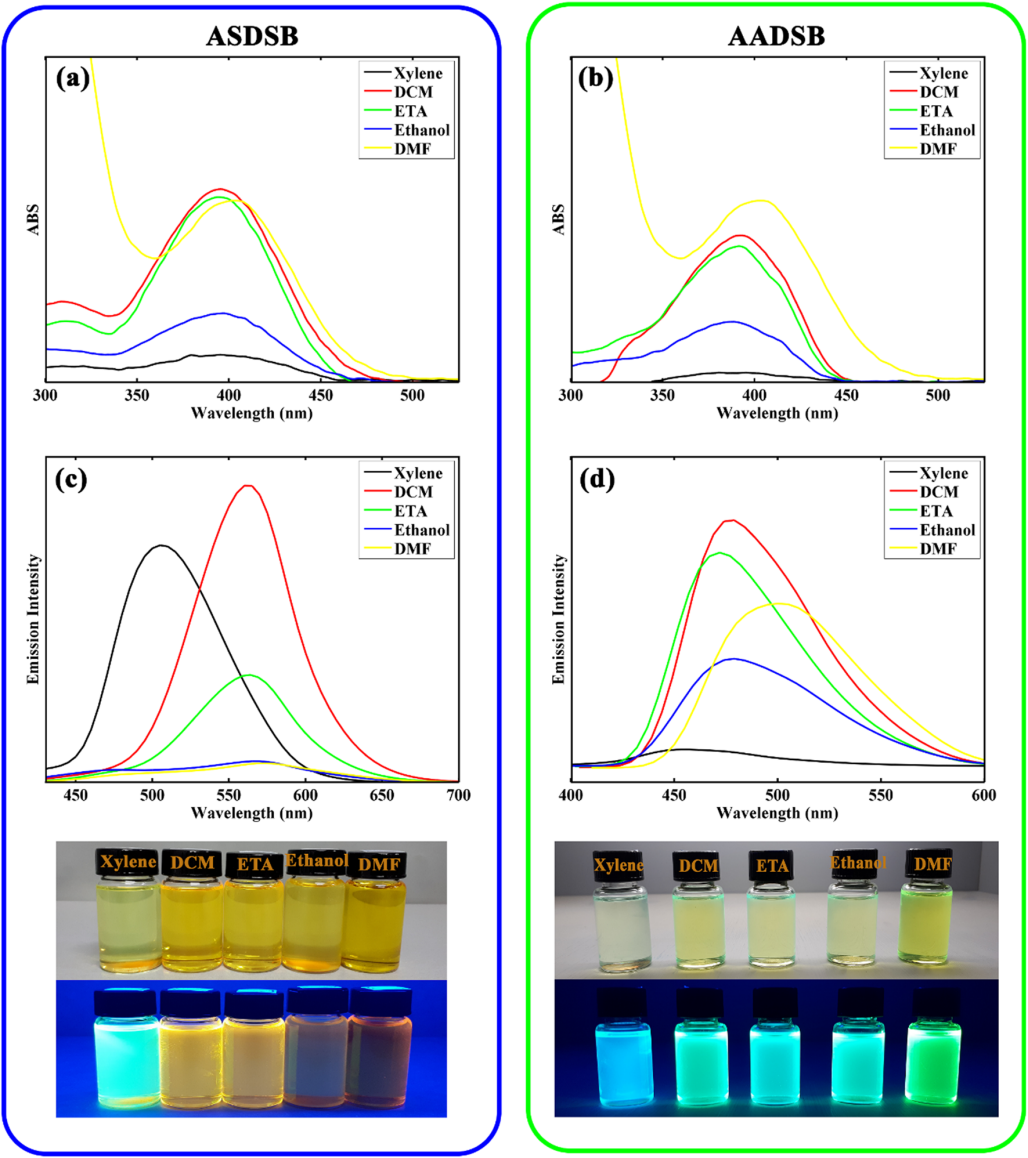
Uv–Vis spectra (**a,b**) and emission spectra (**c,d**) of **ASDSB** and **AADSB** at concentration of 10^–5^ M in different solvents, respectively. The photographs of the chromophores in different solutions (From left to right, xylene, DCM, ETA, ethanol, and DMF) were taken under natural daylight simulator (D65) lamps (top image), and irradiation of A-Class UV lamps (bottom image).

The solvatochromic effect of the chromophores was investigated from the absorption and emission spectra in low to high polarity solvents including xylene, ethyl acetate (ETA), dichloromethane (DCM), ethanol, and dimethylformamide (DMF). The corresponding results are shown in Fig. 2 and Table 1.

**Table 1:** Optical data of the chromophores.

	Solvent	λ_ab_ (nm)	λ_em_ (nm)	Stokes shift (cm^−1^)	ε (L M^−1^ cm^−1^)	E (eV)^a^	Φ^b^
**ASDSB**	Xylene	394	505	5579	9300	2.69	0.73
	DCM	394	563	7619	65,200	2.66	0.13
	ETA	394	554	7330	62,700	2.69	0.1 >
	DMF	401	575	7546	61,300	2.63	0.1 >
	Ethanol	394	570	7837	23,300	2.66	0.1 >
**AADSB**	Xylene	391	451	3403	3400	2.83	0.55
	DCM	394	475	4523	49,600	2.82	0.56
	ETA	392	471	4344	46,000	2.82	0.52
	DMF	404	502	5655	61,200	2.64	0.34
	Ethanol	389	477	4611	20,400	2.82	0.68

^a^Calculated from the absorption spectra by using the empirical formula of E(eV) = hc/λ_onset_ = 1240 (eV nm)/λ_onset_(nm).

^b^ 9,10-Diphenylanthracene was used as standard (Ф = 0.90 in cyclohexane).

The **ASDSB** and **AADSB** compounds showed a broad and structure-less absorption band ranging from 300 to 500 nm with a maximum peak at around 400 nm assigned to the intramolecular charge transfer (ICT) process from the amine and/or sulfonyl group. The absorption band of thechromophores revealed a negligible solvatochromic effect by increasing solvent polarity from low toward high polarity solvents. These observations illustrated that the chromophores exhibit low dipole moments at their ground state. Chromophore **ASDSB** showed a strong fluorescent emission in low to medium polarity solvents (i.e., xylene, dichloromethane (DCM), and ethyl acetate (ETA)) and a weak fluorescent emission in high polar solvents (i.e., ethanol and dimethyl formamide (DMF)). At the same time, **AADSB** showed intense fluorescent emission in all solvents. Chromophore **ASDSB** experienced a bathochromic shift from 505 nm in xylene to 575 nm in DMF, and its fluorescent color intensely changed from greenish-blue to orange. However, this trend was less pronounced for chromophore **AADSB**. This chromophore underwent a bathochromic shift from 451 nm in xylene to 502 in DMF. **AADSB** showed a blue fluorescent color in low polar media that was gradually changed to green with increasing solvent polarity. These results showed that the excited state of the chromophores is more polar and had more ICT characteristics than that of their ground state. The stabilization of the excited state by solvents with more polarity was the reason for the observed solvatochromism^52,53^. The more distinct solvatochromic effect for chromophore **ASDSB** compared to that of chromophore **AADSB** may arise from the difference in their chemical structure. **ASDSB** with strong donor (amine) and acceptor (sulfonyl) substitutions exhibit more intense ICT characters compared to that of **AADSB**. These properties can result in the larger dipole moment and smaller energy bandgap of **ASDSB**. Therefore, the compound at its excited state can easily stabilize by polar solvent molecules interactions. An excellent fluorescence quantum yield (Φ**)** with a value of 0.73 was recorded for the chromophore **ASDSB**. However, the fluorescence was significantly decreased with increasing solvent polarity from xylene to DMF corresponding to the non-radiative relaxation process. As mentioned earlier, polar solvents can stabilize the excited state of the fluorescent compound, narrow the energy gap between its ground and excited state, and induce radiationless decay^54–56^. In the case of chromophore **AADSB**, the fluorescence quantum yield (Φ**)** was found to be high in all solvents with a minimum value of 0.34 in DMF and a maximum value of 0.68 in ethanol.

The solvent effects on fluorescence characteristic of a moleculecan be demonstrated by Lippert–Mataga (LM) formalism^57^:

1 $$\Delta v=\frac{2{\left(\Delta \mu \right)}^{2}}{hc{a}^{3}}\Delta f+constant$$

2 $$\Delta f=\left(\frac{\varepsilon -1}{2\varepsilon +1}\right)-\left(\frac{{n}^{2}-1}{2{n}^{2}+1}\right)$$

**Figure 3:**
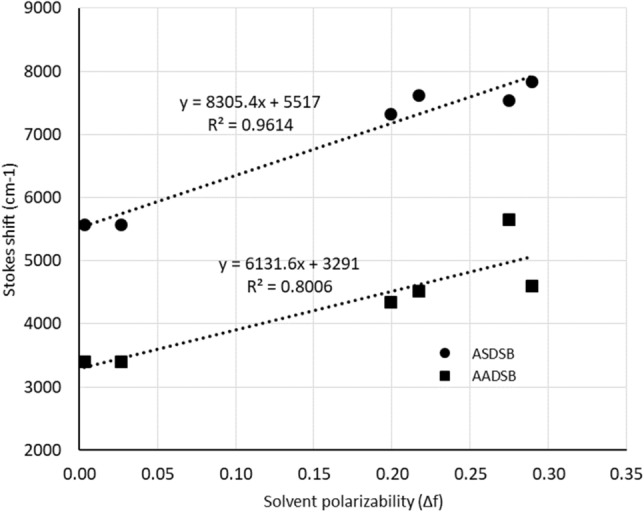
Lippert–Mataga plot for **ASDSB** and **AADSB** shows the correlation between stokes shifts with solvent polarizability characteristics.

In this equation, $\Delta v$, $\Delta \mu$, h, c, a, $\Delta f$, $\varepsilon ,$ and *n* are Stocks shift, the difference of ground state and excited state dipole moments, Planks constant, speed of light, the radius of cavity, solvent polarizability, dielectric constant and refractive index respectively. Equation (2) shows the re-orientation of solvent molecules with dielectric constant (ε) and refractive index (n). The Lippert-Mataga plot is shown in Fig. 3. The slope of the fitted line on the data points indicates the sensitivity of the fluorescence to the solvent polarity. The higher slope of **ASDSB** compared with **AADSB** (8305 and 6131, respectively) shows that the difference of dipole moment of the **ASDSB** in the excited state and ground state is higher than the **AADSB** due to the asymmetric structural design. This result explains the higher stokes shift and lower quantum yields (Φ) of **ASDSB** in polar solvents.

**Figure 4:**
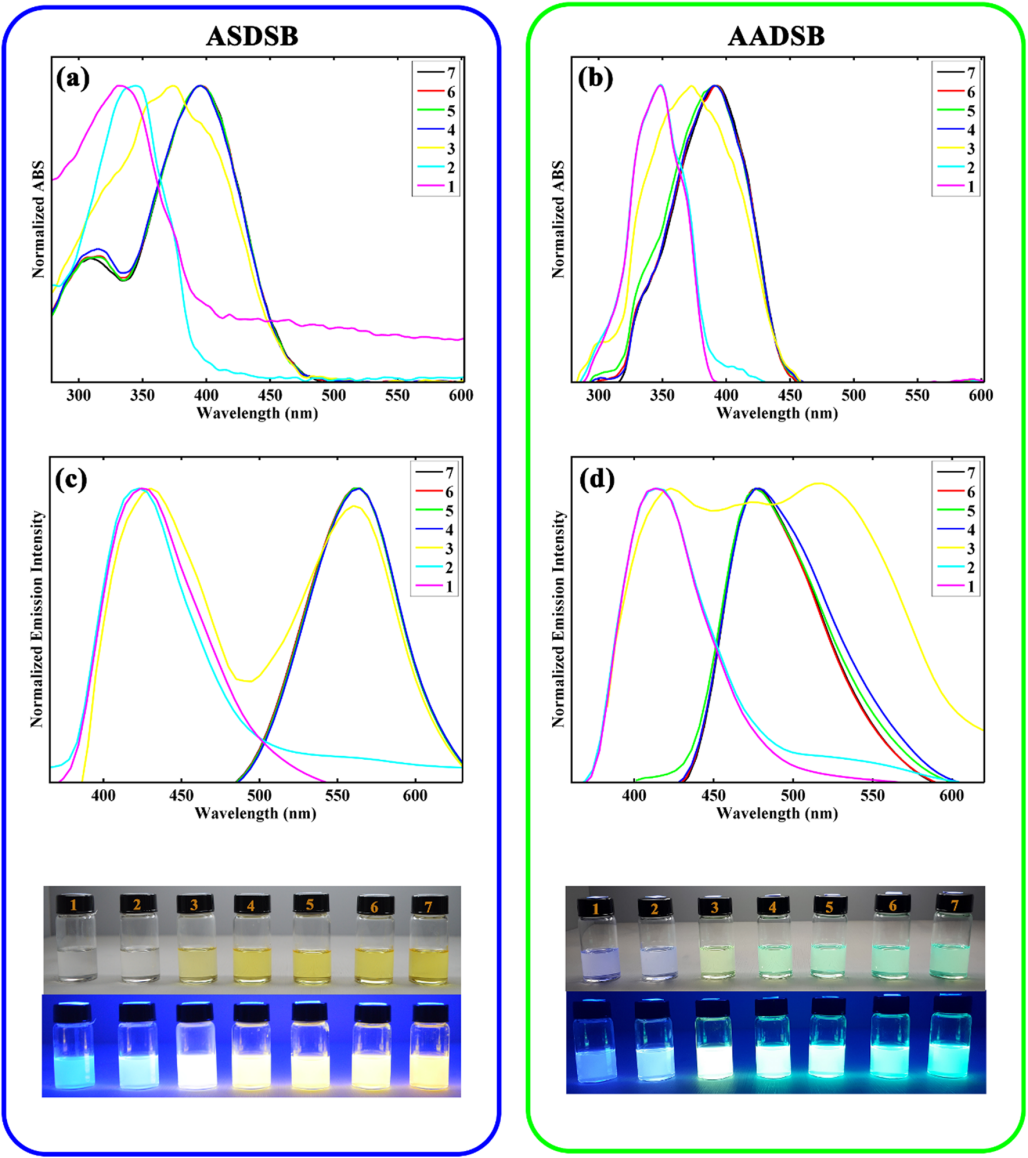
UV–Vis spectra (**a,b**) and emission spectra (**c,d**) of chromophore **ASDSB** and **AADSB** in DCM at a concentration of 10^–5^ M upon change of pH from 1 to 7 by TFA, respectively. The photographs of the solutions (pH increases from left to right) were taken under natural daylight simulator (D65) lamps (top image), and irradiation of A-Class UV lamps (bottom image).

The absorption and emission spectra of **ASDSB** and **AADSB** in DCM upon addition various amounts of trifluoroacetic acid (TFA) were recorded to investigate the effect of protonation on photophysical properties of the chromophores and the results were shown in Fig. 4.

As seen, both absorption and emission spectra of the chromophores showed minor pH responsivity with the variation of pH from 7 to 4. A significant hypsochromic shift was observed for the absorption and emission bands of the chromophores with further decreasing pH from 4 to 1. Correspondingly, the color of the solutions was changed from yellow to colorless for **ASDSB** and green to blue for **AADSB** with decreasing pH. The fluorescent hue was also changed from yellow to blue for **ASDSB** solutions and green to blue for **AADSB** solutions after protonation of the chromophores. The observed hypsochromic shift for chromophores **ASDSB** and **AADSB** were 63 nm (from 397 nm at pH  7 to 334 nm at pH  1) and 44 nm (from 394 nm at pH  7 to 350 nm at pH  1) for their absorption band, respectively. These values were found to be 134 nm (from 563 nm at pH  7 to 429 nm at pH  1), and 57 nm (from 475 nm at pH  7 to 418 nm at pH  1) for their emission band, respectively. These results can be originated from the protonation of the chromophores with increasing acid concentration interrupting the ICT process. In fact, the protonation of the strong electron-donating substitution on the chromophore's molecular structure in acidic conditions can turn the amine group to quaternary ammonium salt as a strong electron-withdrawing group. This procedure weakens electron-donating ability and interrupts the chromophore's push–pull structure, leading to a less ICT character^58,59^.

**Figure 5:**
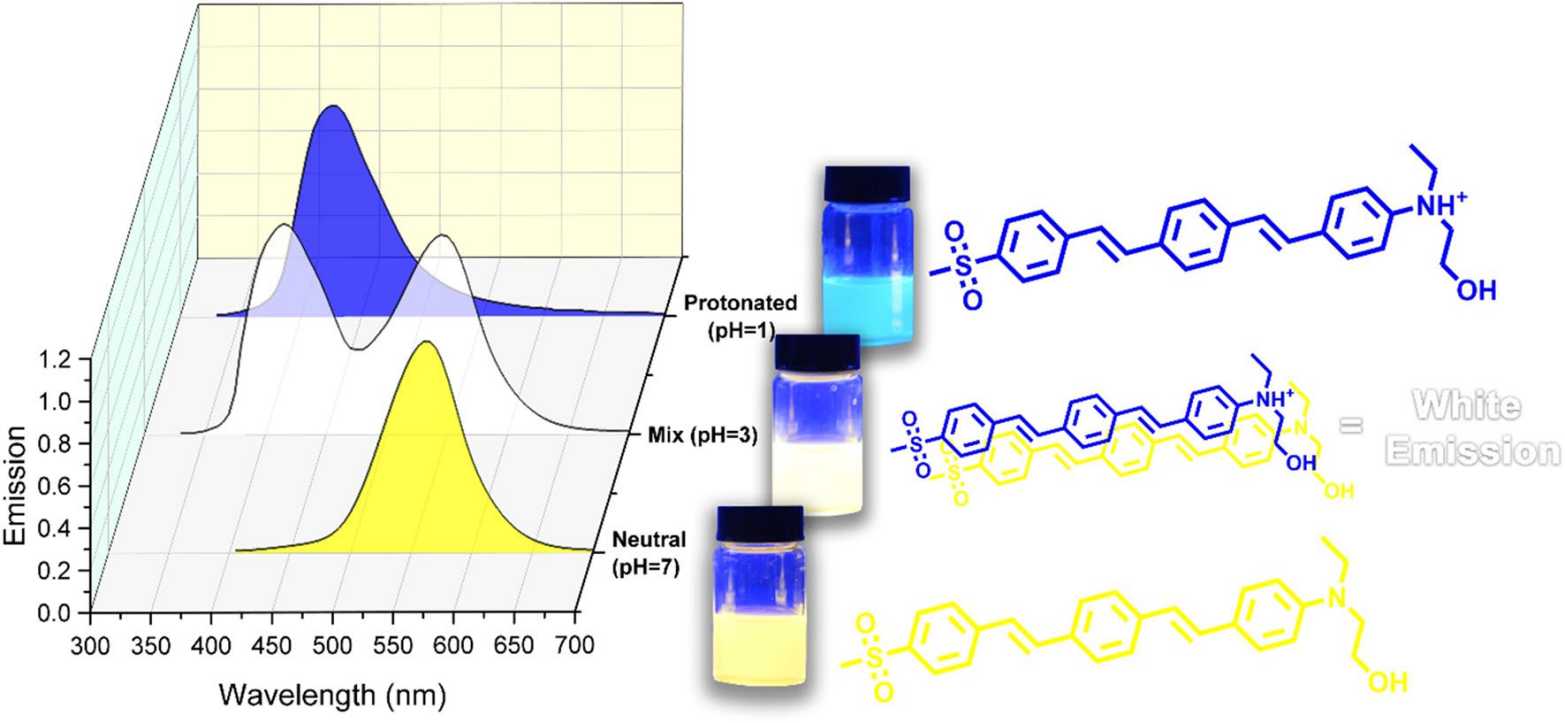
A schematic representation the chemical structure of **ASDSB** beside the emission spectra in both neutral and protonated forms to produce white light emission at pH  3.

The most surprising aspect of the data in Fig. 4 and Table 2 is that the chromophores can illustrate white light emission at pH around 3 with the Commission International de l'Eclairage (CIE 1931) color coordinates of (0.33, 0.32) for **ASDSB** and (0.33, 0.31) for **AADSB**, respectively. This relatively rare phenomenon in fluorescent compounds stemmed from the pH sensitivity of the chromophores. In other words, at a specific pH where the identical amount of neural and protonated forms of the compounds are simultaneously present in their solutions, white light fluorescence can be observed since the emission bands are broad and complementary colors (Fig. 5).

**Table 2:** Optical data of the chromophores at pH  3.

Chromophore	Solvent	(x, y)	Φ
ASDSB	DCM	(0.33, 0.32)	0.31
AADSB	DCM	(0.33, 0.31)	0.54

Another striking observation to emerge from the data was compelling white light quantum yield (Φ**)** with the values of 0.31 and 0.54 for chromophore **ASDSB** and **AADSB**, respectively. It was reported that lighting accounts for almost 20% of the total electricity produced in the globe^60^. Only a small increase in quantum yield of white light emitters could substantially decline electricity consumption. Therefore, the observed results highlighted the potential application of **ASDSB** and **AADSB** as remarkable options in fabricating lighting sources.

**Figure 6:**
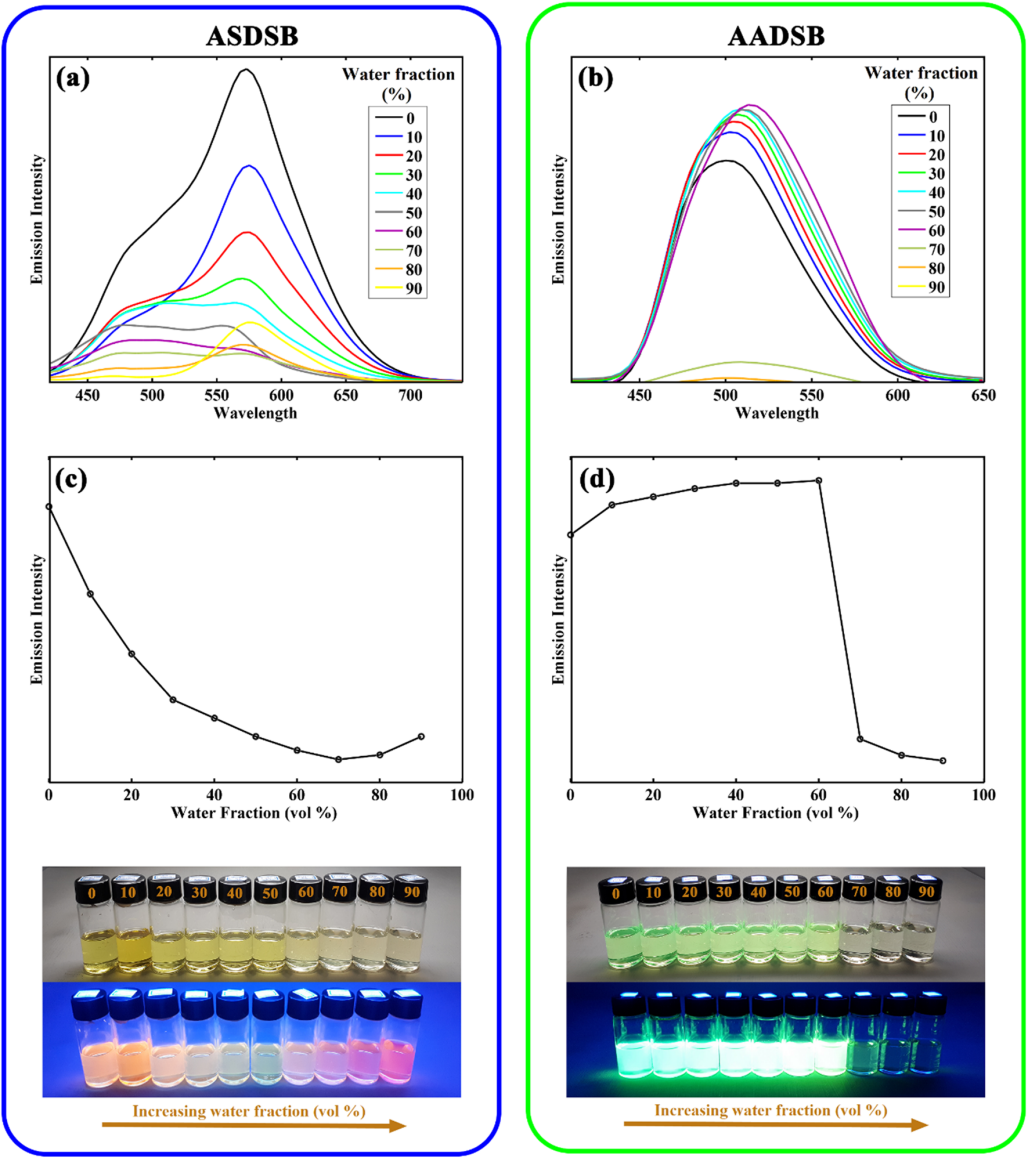
UV–Vis (**a,b**) and emission (**c,d**) spectra of chromophore **ASDSB** and **AADSB** at concentration of 1 × 10^–5^ M in DMF/water mixtures with water fraction from 0 to 90%, respectively. The photographs of the solutions (water fraction increases from 0% (left) to 90% (right)) were taken under natural daylight simulator (D65) lamps (top image), and irradiation of A-Class UV lamps (bottom image).

The emission spectra of the chromophores in the mixture of DMF (good solubility of chromophores) and water (low solubility of chromophores) were obtained to evaluate AIE properties for these compounds. The corresponding results are depicted in Fig. 6.

As can be seen, despite the relative similarity in the molecular structure of chromophore **ASDSB** and **AADSB**, they represented different aggregation characteristics. The main different in the structure of these molecules is the presence of hydroxyl group which is act as both hydrogen donor and hydrogen accepting group as important moiety in the interaction of molecules with the aprotic solvents like water. Therefore, by increasing the fraction of water, the amount of hydrogen bonding between solvent and molecule is enhanced which is directly affects the aggregation of molecule due to the decreasing intramolecular interactions. It should be noted that the sulfone moiety is a hydrogen accepting group and it interact with hydrogens of water molecules resulting in an increasing the positive charge on the sulfur center and finally its act as a stronger accepting group. On the other hand, the amino group acts as a hydrogen acceptor and its interaction with hydrogen of aprotic solvents like water causes a decreasing in the donating power of amino group. Due to these key different in chemical structure of **ASDSB** and **AADSB** molecules their optical behavior and AIE are different. Overall, by increasing the water fraction the electronic nature of the groups, intramolecular polar-polar interactions and solubility of the molecule are changed which are the responsible of both AIE and shifts in emission of the molecules. For chromophore **ASDSB**, upon increasing water fraction from 0 to 70%, the intensity of the emission band at 575 nm was steadily decreased, accompanied by the disappearance of the shoulder at 473 nm. The observed color change from orange to red may be attributed to stabilizing dipole moment at the ground state by increasing solvent interactions such as hydrogen bonding. A slight increase in the emission intensity was observed with further increasing water fractions from 70 to 90%, showing the aggregation-induced emission (AIE) phenomenon for chromophore **ASDSB**. These results may originate from the restriction of fluorophore intramolecular motions due to the formation of molecular aggregations because of low solubility in the mixture solvent and intramolecular interactions. Chromophore **AADSB** displayed a gradual rise in the emission intensity with the concomitant redshift from 502 to 513 nm as the water fraction increased from 0 to 60%. Following the increase of water fraction to 70%, a significant drop in the emission intensity was observed for this sample. Finally, the fluorescence was quenched by further increasing water fraction to 90%, indicative of aggregation-caused quenching (ACQ) for **AADSB**. The increase in the fluorescence of chromophore **ASDSB** (after addition of more than 70% water) and **AADSB** (up to 60% water portion in the solvents mixture) can be explained by the aggregate formation of the chromophores in poor solvents restricting the intermolecular rotations^61,62^. These results were representative of the AIE character in chromophore **ASDSB** and **AADSB**.

### Quantum mechanical studies

Density functional theory (DFT) and time-dependent density functional theory (TD-DFT) were chosen as he most practical ways to investigate the chromophores' electronic structure and vertical transition energies.

**Figure 7:**
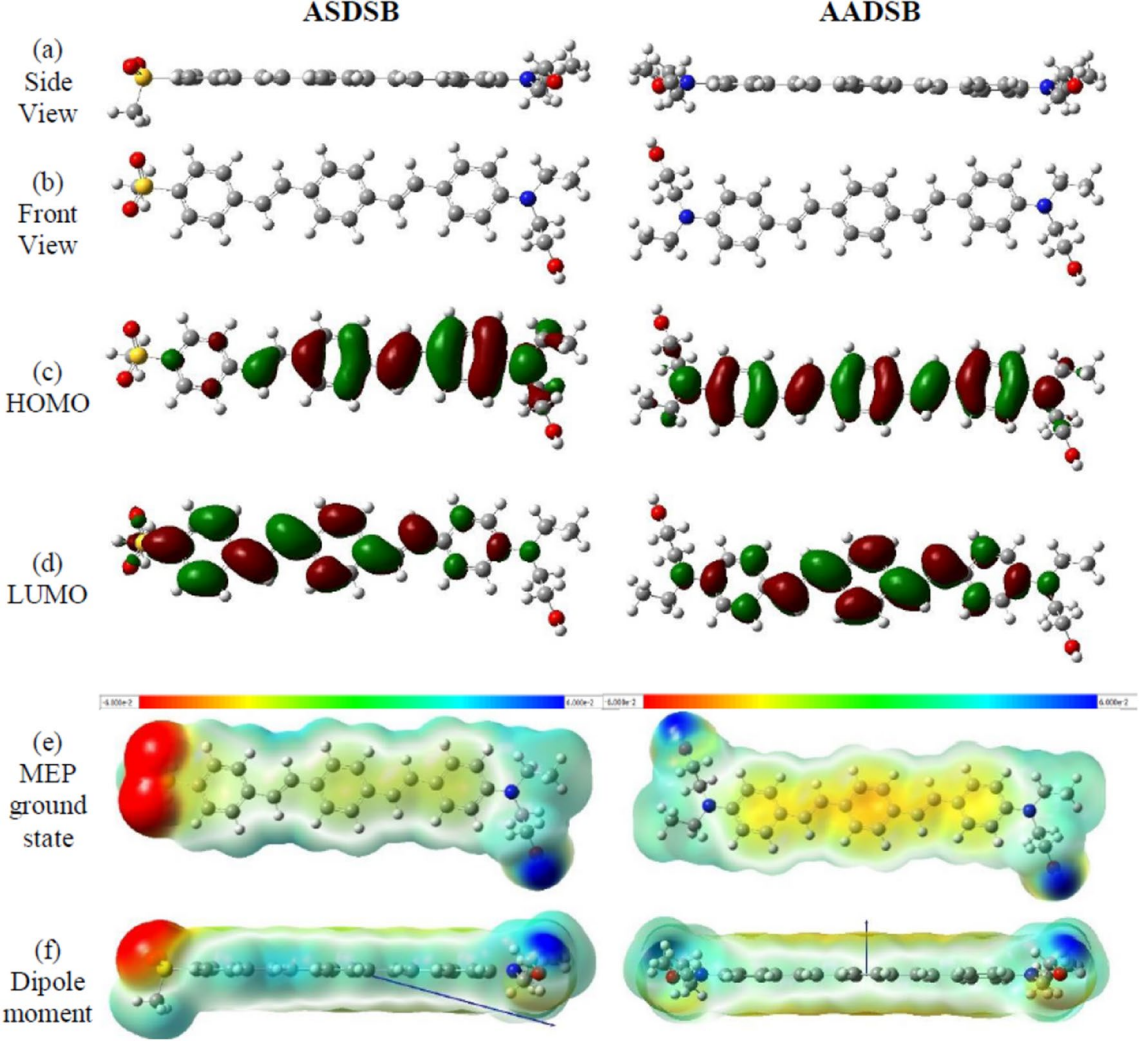
Optimized geometry (**a,b**), HOMO (**b**), LUMO (**c**), MEP maps (**e**), and dipole moment vector illustration (**f**) of **ASDSB** and **AADSB** theoretically modeled at the B3LYP/6–311 + g(2d,p) level.

Figure 7 shows the resulting optimized geometries of **ASDSB** and **AADSB** employingB3LYP/6–311 + g(2d,p) in a DCM solvent environment. The side view of both molecules shows a planar geometry of the three phenyl rings. However, the bulkiness of the amine moiety induces some degree of dihedral twist between the two adjacent phenyl rings. Therefore, in agreement with the higher Stokes shift of **ASDSB** in experiments, the backbone of **ASDSB** is supposed to be more rigid than **AADSB**. The frontier orbitals of the molecules are shown in Fig. 7c,d. The highest occupied molecular orbital (HOMO) of the **ASDSB** is located on amine and the two adjacent phenyl rings. At the same time, the electrons in the HOMO of the **AADSB** are distributed on the entire backbone. While the lowest unoccupied molecular orbital (LUMO) in **AADSB** is somehow localized on the phenyl rings, the LUMO of **ASDSB** is localized on the electron-withdrawing sulfonyl group. The push–pull electronic transfer in **ASDSB** brought a deeper HOMO and LUMO than **AADSB** (Table 3). A most remarkable result from the data is that the HOMO to LUMO transformation in **ASDSB** and **AADSB** is almost different, which suggests different band gaps and absorption wavelengths (λ_max_). Still, according to the experiment and the TD-DFT simulation (Table 3), both molecules show the same λ_max_. On the other hand, molecular electrostatic potential (MEP) surfaces and vectors of dipole moment (Fig. 7e,f) illustrate that the **ASDSB** compared with the **ASDSB**, has a more polar structure. In other words, replacing the electron-withdrawing sulfonyl group with an electron-donating amine grope has no meaningful impact on the absorption characteristics. This is explained by the long system of conjugation, which is brought by the sequence of stilbenes in the backbone of the molecules. Putting the donor and acceptor in such a far distance led to the same length of conjugation for both molecules and diminishing the push–pull effect on the absorption wavelength, yet different solvatochromic and fluorescence characteristics, as seen in experiments and Lipppert–Mataga study.

**Table 3:** Ground state (DFT) and excited state (TD-DFT) calculated parameters of **ASDSB** and **AADSB.**

	**ASDSB**	**AADSB**
**Ground state**		
HOMO	−5.16	−4.90
LUMO	−2.39	−1.92
Gap	2.78 (2.66)	2.98 (2.84)
Dipole moment	11.7	3.1
**Excited-state**		
λ_max_ (nm)	404 (394)	401 (394)
Oscillator strength	2.3772	2.5841
Major contributions	HOMO → LUMO (78%)	HOMO → LUMO (89%)

## Conclusion

In conclusion, two stilbene-based chromophores were introduced, which showed yellow and green emission fluorescence in their neutral form and blue fluorescence color with a strong hypsochromic shift in the protonated forms. These chromophores showed WLE at pH  3 due to the simultaneous presence of the neutral and protonated chromophores, representing pH-responsive fluorescence. Remarkable white light quantum yields (Φ) were observed for producing white light-emitting devices in these chromophores, suggesting a high potential application of the compounds in the preparation of high-performance lighting devices.

## Experimental

### General

Chemicals were purchased from Fluka and Aldrich companies and used without further purification. The known products were characterized by comparing their spectral and physical data with those reported in the literature. ^1^H NMR (250 MHz) and ^13^C NMR (62.5 MHz), spectra were recorded on a Brucker (250 MHz) Avance DRX. FT-IR spectroscopy (Shimadzu FT-IR 8300 spectrophotometer), were employed for the characterization of the products. Melting points were determined in open capillary tubes in a Buchi melting point B-545. The reaction monitoring was accomplished by TLC on silica gel PolyGram SILG/UV254 plates. Column chromatography was carried out on columns of silica gel 60 (70–230 mesh).

### Procedure for the synthesis of compounds ASDSB and AADSB

1-(Methylsulfonyl)-4-(4-vinylstyryl)benzene (**C**). A sealed Schlenk tube was charged with1-bromo-4-methanesulfonyl-benzene (**A**; 5 mmol, 1.17 g), and K_2_CO_3_ (10.0 mmol, 1.4 g), Pd(OAc)_2_ (1.2 mol%, 14 mg), DPEPhos ligand (2.4 mol%, 65 mg) and it was evacuated and backfilled with argon. Then 1,4-divinyl-benzene (**B**; 5 mmol, 0.6 mL) and 5 mL of dry DMF was added to the reaction mixture under fellow of argon and tube was sealed with a screw-cap and the resulting mixture was heated in an oil bath at 120 °C for 6 h. To obtain the pure product its was purified by column chromatography (hexane/ethyl acetate: 10/1) (1.04 g, 73%). Yellow solid; mp 182.7 °C. IR (KBr): 3448, 3016, 1589, 1412, 1311, 1149, 957, 833, 764, 448 cm^-1^. ^1^H-NMR (250 MHz, CDCl_3_/TMS) δ (ppm): 3.07 (s, 3H), 5.29 (d, *J* = 11.0 Hz, 1H), 5.80 (d, *J* = 17.5 Hz, 1H), 6.73 (dd, *J* = 17.6, 10.7 Hz, 1H), 7.12 (d, *J* = 16.5, 1H), 7.24 (d, *J* = 16.5 Hz, 1H), 7.43 (d, *J* = 8.3 Hz, 2H), 7.51 (d, *J* = 8.2 Hz, 2H), 7.67 (d, *J* = 8.5 Hz, 2H), 7.92 (d, *J* = 8.5 Hz, 2H). ^13^C-NMR (62.5 MHz, CDCl_3_/TMS) δ (ppm): 44.6, 114.5, 126.3, 126.7, 127.0, 127.1, 127.9, 132.2, 135.7, 136.2, 137.9, 142.8. Anal. Cal. C_17_H_16_O_2_S (284.4): C, 71.80; H, 5.67; O, 11.25; S, 11.28; found: C, 71.85; H, 5.71.

2–4- 2-{Ethyl-[4-(2-{4-[2-(4-methanesulfonyl-phenyl)-vinyl]-phenyl}-vinyl)-phenyl]-amino}-ethanol (**ASDSB**). A sealed Schlenk tube was charged with compound **C** (1 mmol, 0.29 g), 2-[Ethyl-(4-iodo-phenyl)-amino]-ethanol (**D**; 1 mmol, 0.29 g), and K_2_CO_3_ (2.5 mmol, 0.35 g), Pd(OAc)_2_ (1.2 mol %, 2.7 mg), DPEPhos ligand (2.4 mol %, 13 mg) and it was evacuated and backfilled with argon. Then 5 mL of dry DMF was added to the reaction mixture under fellow of argon and tube was sealed with a screw-cap and the resulting mixture was heated in an oil bath at 120 °C for 12 h. After completion of the reaction, the mixture was filtered (in hot form) and the remaining solid was washed with DMF (2 mL). Subsequently, water (10 mL) was added to the solution in order to precipitate product. The obtained solid was purified by column chromatography (hexane/ethyl acetate: 10/2) to obtain the pure product (0.4 g, 90%). Orange solid; mp 275.5 °C.^1^ IR (KBr): 3300, 3000, 1600, 1590, 1510, 1400, 1350, 1290, 1180, 1130, 1080, 1060, 830, 820, 760, 550 cm^−1^. ^1^H-NMR (250 MHz, DMSO-d_6_/TMS) δ (ppm): 1.07 (t, *J* = 6.8 Hz, 3H), 3.20 (s, 3H), 3.32–3.51 (m, 6H), 4.72 (s, 1H), 6.66 (d, *J* = 8.6 Hz, 2H), 6.93 (d, *J* = 16.4 Hz, 2H), 7.15 (d, *J* = 16.4 Hz, 2H), 7.36–7.42 (m, 2H), 7.48–7.62 (m, 6H), 7.81–7.90 (m, 2H). Anal. Cal. C_27_H_29_NO_3_S (447.6): C, 72.45; H, 6.53; N, 3.13; O, 10.72; S, 7.16; found: C, 72.51; H, 6.58; N, 3.19.

2-[Ethyl-(4-{2-[4-(2-{4-[ethyl-(2-hydroxy-ethyl)-amino]-phenyl}-vinyl)-phenyl]-vinyl}-phenyl)-amino]-ethanol (**AADSB**). A sealed Schlenk tube was charged with 2-[Ethyl-(4-iodo-phenyl)-amino]-ethanol (**D**; 2 mmol, 0.60 g), K_2_CO_3_ (5 mmol, 0.68 g), Pd(OAc)_2_ (1.2 mol%, 5.5 mg), DPEPhos ligand (2.4 mol%, 26 mg) and it was evacuated and backfilled with argon. Then 1,4-divinyl-benzene (**B**; 1 mmol, 0.12 mL) and 6 mL of dry DMF were added to the reaction mixture under the argon atmosphere. The tube was sealed with a screw-cap, and the resulting mixture was heated in an oil bath at 120 °C for 6 h. The reaction was followed by TLC. After completion of the reaction, the mixture was cooled down to room temperature and filtered. The remaining solid was washed with dichloromethane (3 × 5 mL) to separate the catalyst. After the extraction of dichloromethane from water, the organic extract was dried over Na_2_SO_4_. The products were purified by column chromatography (hexane/ethyl acetate: 10/2) to obtain the pure product (0.4 g, 88%). Yellow solid; mp 216.5 °C. IR (KBr): 3389, 2922, 1601, 1520, 1360, 1267, 1180, 1051, 964, 823, 550 cm^−1^. ^1^H-NMR (250 MHz, DMSO-d_6_) (δ ppm): 1.04–1.14 (m, 6H), 3.31–3.53 (m, 12H), 4.70 (m, 2H), 6.55–6.67 (m, 4H), 6.85–7.10 (m, 8H), 7.22–7.66 (m, 4H). *m/z* (%): 456 (94.5%, (M)^+^). Anal. Cal. C_30_H_36_N_2_O_2_ (456.6): C, 78.91; H, 7.95; N, 6.13; O, 7.01; found: C, 78.98; H, 7.99; N, 6.18.

### Theoretical calculations

The geometry of the molecules in the ground state was optimized by the first-principles density functional theory (DFT). The calculations carried by B3LYP functional^63^ and 6–311 + g(2d,p)as the basis set. The most stable geometry was found by examining different isomers and configurations and examining vibrational frequency calculation. The excited state of the molecules was simulated by time-dependent density functional theory (TD-DFT). The model was the Coulomb attenuating employing the B3LYP hybrid functional (CAM-B3LYP)^64^ and the same basis-set as the DFT method. The polarizable continuum model with the integral equation formalism (IEFPCM) was chosen to model the dichloromethane (DCM) as solvent. Calculations were carried out using Gaussian 09^65^.
